# Utility of Harmonisation for Fixel‐Based Metrics in Travelling Subjects and Alzheimer's Disease Data

**DOI:** 10.1002/hbm.70408

**Published:** 2025-11-12

**Authors:** Rui Zou, Koji Kamagata, Remika Mito, Kaito Takabayashi, Christina Andica, Wataru Uchida, Sen Guo, Takafumi Kitagawa, Shohei Fujita, Akiko Uematsu, Norihide Maikusa, Shinsuke Koike, Shigeki Aoki, Alzheimer's Disease Neuroimaging Initiative

**Affiliations:** ^1^ Department of Radiology Juntendo University Graduate School of Medicine Tokyo Japan; ^2^ Department of Data Science Juntendo University Graduate School of Medicine Tokyo Japan; ^3^ Department of Psychiatry The University of Melbourne Melbourne Victoria Australia; ^4^ Florey Department of Neuroscience and Mental Health The University of Melbourne Melbourne Victoria Australia; ^5^ Faculty of Health Data Science, Juntendo University Urayasu Chiba Japan; ^6^ Laboratory for Brain Connectomics Imaging, RIKEN Center for Biosystems Dynamics Research Kobe Hyogo Japan; ^7^ Center for Evolutionary Cognitive Sciences, Graduate School of Art and Sciences The University of Tokyo Tokyo Japan

**Keywords:** Alzheimer's disease, ComBat harmonisation, fixel‐based analysis, multi‐centre dMRI studies, travelling subject

## Abstract

Fixel‐based analysis (FBA) is an advanced diffusion MRI analysis technique that facilitates the evaluation of white matter microstructure within ‘fixels’ (specific fibre populations within a voxel). In recent years, FBA has gained prominence for its ability to better characterise fibre tract‐specific changes than the more conventional diffusion MRI approaches and has shown promise in elucidating the pathophysiology of psychiatric and neurological diseases. However, FBA has been predominantly limited to single‐centre studies, minimising the generalisability of the technique. In this study, the popular ComBat harmonisation technique was adapted for whole‐brain FBA of diffusion MRI data. The study evaluates the effectiveness of ComBat in harmonising FBA metrics of fibre density, fibre cross‐section and the combined metric of fibre density and cross‐section in a large travelling subject dataset (*n* = 49, scan = 162). Participants were scanned across multiple centres, using different scanner models and imaging protocols, and FBA metrics were compared under these varying conditions before and after harmonisation. In addition, the impact of ComBat harmonisation on disease‐related findings was evaluated in an independent multi‐centre Alzheimer's disease (AD) dataset, by comparing the same fixel‐based measures in patients with AD (*n* = 27) to those in cognitively normal control participants (*n* = 29) before and after ComBat harmonisation. We demonstrated that ComBat harmonisation effectively mitigated variability across scanner sites, scanner models, and protocols, in the travelling subject dataset, thus enhancing the comparability of FBA metrics. Notably, ComBat harmonisation improved the detection of AD‐related changes in the fornix, a critical white matter tract associated with cognitive function, and strengthened the correlations between FBA metrics and cognitive scores. These results underscore the potential of ComBat harmonisation in enhancing the reliability of multi‐centre neuroimaging studies, supporting the use of harmonisation techniques for accurate detection of disease‐specific changes in neurodegenerative conditions. The ability to perform ComBat harmonisation within the whole‐brain FBA pipeline may help further this fibre‐specific technique into large‐scale multi‐centre studies.

## Graphical abstract

ComBat harmonisation effectively reduces measurement bias in fixel‐based diffusion MRI metrics across multiple sites, scanner models, and imaging protocols. This enables improved detection of Alzheimer's disease‐related changes in white matter microstructure. The approach enhances both the reliability and scalability of multi‐centre neuroimaging studies.

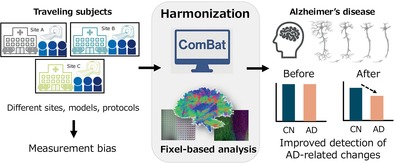

## 1 Introduction

Advanced diffusion‐weighted magnetic resonance imaging (dMRI) analysis techniques, including fixel‐based analysis (FBA) (Raffelt et al. 2017), offer the possibility of examining subtle changes to white matter (WM) fibre pathways in healthy and diseased individuals. FBA, based on modelling dMRI data using constrained spherical deconvolution (CSD), enables the characterisation of multiple fibre orientations within a single voxel, providing refined information about WM microstructure at the fixel level. In this context, a ‘fixel’ refers to a specific fibre population within a voxel (Raffelt et al. 2015; Dhollander and Connelly 2016; Tournier et al. 2007; Jeurissen et al. 2014). FBA typically quantifies three fixel‐based metrics: fibre density (FD), fibre cross‐section (FC) and the combined metric of fibre density and cross‐section (FDC). These metrics provide information on WM microstructural properties. Hence, FBA is particularly valuable for evaluating WM integrity across a range of neurological and psychiatric disorders, including Alzheimer's disease (AD) (Mito et al. 2018; Wei et al. 2024; Ahmadi et al. 2024), Parkinson's disease (Andica et al. 2023; Li et al. 2020), schizophrenia (Kristensen et al. 2023) and depression (Lyon et al. 2019). However, research on FBA has hitherto been limited to small‐sample, single‐centre studies. Expanding FBA research beyond single‐centre studies could help to identify more reproducible and reliable findings of fibre tract‐specific WM changes that characterise various clinical conditions (Marek et al. 2022). To this end, multi‐centre dMRI studies have garnered attention, given their capability to identify subtle changes to WM microstructure with high statistical power, being increasingly utilised in clinical research (Sprague et al. 2009; Pohl et al. 2016; Schilling et al. 2022; Mohammad et al. 2024). Nevertheless, dMRI metrics among different centres have variations, often referred to as measurement bias, including hardware differences (e.g., magnetic field inhomogeneities), reconstruction algorithms and acquisition parameters (e.g., *b* values, echo time and voxel size) (Afzali et al. 2021), which can result in significant inconsistencies. Indeed, studies employing diffusion tensor imaging (DTI) and Neurite Orientation Dispersion and Density Imaging (NODDI), which report inter‐site variability of up to 5%–10% in metrics like fractional anisotropy and intracellular volume fraction (ICVF) (Andica et al. 2020; Chung et al. 2016). Notwithstanding, the variability of FBA metrics across different sites and acquisition settings remains poorly characterised, due to its relative novelty relative to other dMRI analysis techniques.

Therefore, accounting for this variability is crucial when analysing multi‐centre dMRI datasets to ensure accurate and reliable results. To address these challenges, harmonisation methods employing statistical or mathematical approaches have been developed to mitigate measurement bias while retaining biological information (Yamashita et al. 2019; Pinto et al. 2020; Pomponio et al. 2020). One such harmonisation technique is the Combined Association Test (ComBat), originally developed to correct batch effects in genomics (Johnson et al. 2007) and subsequently adapted for neuroimaging.

ComBat has been successfully used to harmonise various imaging‐derived metrics, such as cortical thickness from structural MRIs (Fortin et al. 2018), as well as DTI and NODDI (Fortin et al. 2017; Saito et al. 2024). These studies have shown reductions in variability between diffusion metrics across sites after ComBat harmonisation and the maintenance of associations with biological variables such as age and sex. Thus, applying ComBat harmonisation to fixel‐based metrics derived from multi‐centre large‐scale data would be beneficial for further use in disease‐related datasets, such as those involving AD.

In this study, we applied ComBat harmonisation on FBA metrics derived from a multi‐centre dMRI dataset. Our goal was to first evaluate the effectiveness of ComBat in reducing measurement bias derived from different sites, scanner models and protocols on FBA metrics using data with ground‐truth comparison (travelling subject [TS] data, where the same individuals were scanned using different scanners and using different imaging protocols). We aimed to evaluate the utility of ComBat harmonisation for FBA metrics when performing group comparisons between healthy participants and AD patients.

## 2 Materials and Methods

### 2.1 Study Design and Grouping of TS Data

The TS dataset from the Brain/MINDS Beyond Human Brain MRI Project (https://brainminds‐beyond.jp/) was used in this study. This dataset includes 75 participants who were scanned across 13 sites using 15 different MRI scanners, yielding approximately 450 scans. This project followed a ‘hub‐and‐spoke’ design, where each participant was scanned at five to six different sites (Koike et al. 2021).

To evaluate the effectiveness of ComBat harmonisation in reducing measurement bias introduced by site differences, scanner models and imaging protocols, we categorised the data into four experimental groups:

1. Scan–rescan group: Participants were scanned twice at the same site using the same MRI scanner model and protocol to assess the stability of FBA metrics.
2. Site–difference group: Participants were scanned at two different sites using the same MRI scanner model and protocol to evaluate the impact of site differences on FBA metrics.
3. Scanner model–difference group: Participants were scanned using two different MRI scanner models at the same site, with the same protocol, to determine the effect of scanner model variation on FBA metrics.
4. Protocol–difference group: Participants were scanned using two different imaging protocols at the same site and with the same MRI scanner model to assess the impact of protocol variations.

We extracted all relevant data fulfilling the criteria for these four groups, yielding 162 scans from 49 participants across six sites, utilising three different scanner models and two imaging protocols (Table 1).

**TABLE 1:** Summary of participant data and scanning conditions.

Group	Participants (*n*)	Age (mean ± SD)	Sex (M/F)	Scans (*n*)	Sites (*n*)	Scanner (*n*)	Protocols (*n*)
Scan‐rescan	23	31.8 ± 2.1	13/10	46	6	1	1
Site‐difference	10	29.9 ± 2.0	4/6	20	2 (UTI, UTK)	1	1
Scanner model‐difference	23	30.2 ± 1.8	13/10	46	1	2 (Siemens Prisma fit vs. Siemens Verio)	1
Protocol‐difference	31	33.9 ± 1.6	19/12	62	1	1	2 (HARP vs. CRHD)

Abbreviations: CRHD, Connectomes Related to Human Disease protocol; F, female; HARP, harmonised MRI imaging protocol; M, male; SD, standard deviation; UTI, University of Tokyo International Research Center for Neurointelligence; UTK, Center for Evolutionary Cognitive Sciences at the University of Tokyo.

### 2.2 Participants With AD Collected From Alzheimer's Disease Neuroimaging Initiative (ADNI)

Data used in the preparation of this article were obtained from the ADNI database (adni.loni.usc.edu). The ADNI was launched in 2003 as a public–private partnership led by Principal Investigator Michael W. Weiner, MD. The ADNI primarily aims to test whether serial MRI, positron emission tomography, other biological markers and clinical and neuropsychological assessment can be combined to measure the progression of mild cognitive impairment and early ad (Petersen et al. 2010). The participants of this study included individuals diagnosed with AD and cognitively normal (CN) controls, aged 55–90 years, who were enrolled in the ADNI‐3 study. The inclusion criteria required participants to have completed ≥ 10 years of education and to be free of any neurological disease other than AD. Specific group requirements were as follows: (1) The AD group: Participants met the diagnostic criteria for AD as defined by the National Institute of Neurological and Communicative Disorders and Stroke and the AD and Related Disorders Association (NINCDS‐ADRDA) (McKhann et al. 2011). (2) The CN group: Participants were classified as CN at baseline and during follow‐up assessments. Both AD and CN participants underwent MRI scans using one of six scanner models: Siemens Prisma, Siemens Prisma_fit, GE DISCOVERY MR750, GE DISCOVERY MR750w, Philips Ingenia or Siemens Skyra. Each scanner model was used for at least two participants to ensure consistency across groups. Participants in the AD and CN groups were matched for age and sex to minimise confounding effects. Participants with poor image quality or those for whom pre‐processing could not be performed were excluded from further analysis. Detailed participant information is presented in Table 2.

**TABLE 2:** Demographics of participants.

	CN	AD	*p*
No. of participants	29	27	—
Age (mean ± SD)	72.5 ± 6.0	72.3 ± 8.5	0.91
Sex (M/F)	16/13	17/10	0.56
MMSE (mean ± SD)	29.0 ± 1.0	22.6 ± 2.3	**< 0.0001**
MoCA (mean ± SD)	26.4 ± 2.1	16.9 ± 3.4	**< 0.0001**
No. of scanners	6	6	—

*Note:* Bold indicates statistical significance.

Abbreviations: AD, Alzheimer's disease; CN, cognitively normal; MMSE, Mini‐Mental State Examination; MoCA, Montreal Cognitive Assessment; SD, standard deviation.

### 2.3 MRI Acquisition

Diffusion MRI data for TS participants were acquired using 3‐Tesla MRI scanners, including the Siemens Prisma Fit, Siemens Prisma or Siemens Verio models. All MRI scans were performed using a 32‐channel head coil, employing a single‐shot spin‐echo echo‐planar imaging sequence with monopolar diffusion gradients. Acquisition parameters specific to each scanner model and acquisition protocol are detailed in Table 3.

**TABLE 3:** TS dMRI acquisition parameters.

	Scanner model‐difference		Protocol‐difference	
Scanner model	Siemens Prisma fit	Siemens Verio	Siemens Prisma	Siemens Prisma
Protocol	HARP	HARP	CRHD	HARP
Repitition time (ms)	3600	3600	3230	3600
Echo time (ms)	79	89	89.2	79
Flip angle (deg)	90	90	78	90
Gradient strength (mT/m)	80	45	80	80
Field of view (mm)	204 × 204 × 144	204 × 204 × 144	210 × 210 × 138	204 × 204 × 144
Matrix size	120 × 120	120 × 120	140 × 140	120 × 120
No. of slices	84	84	92	84
Voxel size (mm)	1.7 × 1.7 × 1.7	1.7 × 1.7 × 1.7	1.5 × 1.5 × 1.5	1.7 × 1.7 × 1.7
Partial Fourier	6/8	6/8	0.75	6/8
Multi‐band factor	3	3	4	3
*b* values (s/mm)	0/700/2000	0/700/2000	0/1500/3000	0/700/2000
No. of directions (AP)	5/16/32	7/20/40	7/47/46	5/16/32
No. of directions (PA)	6/16/32	8/20/40	7/47/46	6/16/32
Scan time (min:s)	7:02	9:44	11:18	7:02

For participants from ADNI, dMRI data were collected using various MRI scanner models, including Siemens Prisma, Siemens Prisma Fit, GE DISCOVERY MR750, GE DISCOVERY MR750w, Philips Ingenia and Siemens Skyra. Acquisition parameters for these scanner models and additional details (Table 4) on imaging protocols can be found on the ADNI website: http://adni.loni.usc.edu/methods/documents/mri‐protocols/.

**TABLE 4:** ADNI dMRI acquisition parameters.

Scanner model	Siemens Prisma	Siemens Prisma_fit	Siemens Skyra	Philips Ingenia			GE DISCOVERY MR750		GE DISCOVERY MR750w
Protocol	Axial DTI	Axial DTI	Axial DTI	Axial DTI	Axial DTI SENSE	WIP Axial DTI	ADNI3_GE_3T_24x	ADNI3 Basic Human Protoc	ADNI3_Study_Human_GE_3T
Repitition time (ms)	7200	7200	9600	10,948	10,899	11,199	7800	7800	15,781
Echo time (ms)	56	56	82	99	99	101	61	60	76
Gradient strength (mT/m)	80	80	80	45	45	45	50	50	50
Field of view (mm)	232 × 232 × 160	232 × 232 × 160	232 × 232 × 160	256 × 256 × 160	256 × 256 × 160	256 × 256 × 160	232 × 232 × 158	232 × 232 × 160	232 × 232 × 160
Matrix size	116 × 116	116 × 116	116 × 116	128 × 128	128 × 128	128 × 128	256 × 256	256 × 256	256 × 256
No. of slices	80	80	80	80	80	80	79	80	80
Voxel size (mm)	2 × 2 × 2	2 × 2 × 2	2 × 2 × 2	2 × 2 × 2	2 × 2 × 2	2 × 2 × 2	0.9 × 0.9 × 2	0.9 × 0.9 × 2	0.9 × 0.9 × 2
Bandwidth (Hz/Px)	2270	2270	1540	1866	1914	1715	1953	1953	1935
Echo spacing (ms)	0.57	0.57	0.36	0.35	0.35	0.37	0.15	0.15	0.17
*b* values (s/mm)	0/1000	0/1000	0/1000	0/1000	0/1000	0/1000	0/1000	0/1000	0/1000
No. of directions	7/48	7/48	7/48	4/32	1/32	4/32	7/48	7/48	4/32
Scan time (min:s)	10:48	12:42	14:52	11:06	9:44	10:59	15:24	12:11	13:24

### 2.4 MRI Data Processing

The acquired dMRI data were pre‐processed using MRtrix3 (version 3.0.4 and 3Tissue_v5.2.9) and FSL 6.0.1, following the best practices established in recent studies (Smith et al. 2004; Tournier et al. 2019). Pre‐processing and FBA were performed separately for the two datasets due to differences in acquisition protocols.

#### 2.4.1 TS Dataset Processing

Pre‐processing of TS data included denoising (Veraart et al. 2016), gibbs ringing removal (Kellner et al. 2015), correction for eddy current and subject motion (Smith et al. 2004), bias field correction and up‐sampling to an isotropic voxel size of 1.5 mm (Dhollander and Connelly 2016). Response functions for WM, grey matter (GM) and cerebrospinal fluid (CSF) were estimated using the Dhollander algorithm and averaged across subjects (Jeurissen et al. 2014). Fibre orientation distributions (FODs) were reconstructed using Multi‐Shell Multi‐Tissue CSD (Jeurissen et al. 2014).

To achieve spatial correspondence, a population‐specific FOD template was generated using an iterative registration and averaging approach using FOD images from a randomly selected subset of 40 participants (Raffelt et al. 2011, 2012). Each subject's FOD image was then registered to the template via a FOD‐guided non‐linear registration (Raffelt et al. 2011). Subject FODs were segmented into fixels, followed by reorientation and correspondence mapping to ensure accurate alignment across individuals. Whole‐brain tractography was performed on the FOD template to derive a fixel–fixel connectivity matrix, which was applied for connectivity‐based smoothing of each FBA metric to reduce reconstruction biases by using connectivity‐based fixel enhancement (Raffelt et al. 2015). This framework enabled group‐wise comparison of fixel‐based metrics, including FD, log‐transformed FC (logFC) and FDC (Raffelt et al. 2017).

#### 2.4.2 ADNI Dataset Processing

Pre‐processing of ADNI data followed the same pipeline as described for the TS dataset, with minor adaptations for the single‐shell acquisition protocol. Specifically, eddy‐current distortions and subject motion were corrected using FSL's *eddy* without the additional *topup* step. Response functions for WM, GM and CSF were estimated using the Dhollander algorithm and averaged across subjects. FODs were then reconstructed using Single‐Shell 3‐Tissue CSD (Dhollander et al. 2016, 2019).

To achieve spatial correspondence, a population‐specific FOD template was generated from a randomly selected subset of 40 subjects (20 ad patients and 20 CN controls) using an iterative registration and averaging approach (Raffelt et al. 2011). Subject FODs were segmented into fixels with correspondence established across individuals, and a template‐based tractogram was used for connectivity‐informed smoothing of FD, logFC and FDC (Raffelt et al. 2015), which were then compared group‐wise using the same procedures as applied to the TS dataset.

### 2.5 ComBat Harmonisation

ComBat harmonisation is an extension of the general linear model (GLM) that uses an empirical Bayesian framework to remove non‐biological variability while retaining biological differences in the data (Johnson et al. 2007). We harmonised FBA metrics using the ComBat framework (Fortin et al. 2018, 2017), adapted to the fixel image format in MRtrix3. In this approach, each template‐defined fixel is treated as an independent feature, and harmonisation is performed separately for each FBA metric.

All subjects' FOD images were nonlinearly registered to the population FOD template, from which a common fixel mask was defined (Jeurissen et al. 2014). This procedure ensured that each subject's fibre populations were mapped onto the same set of template fixels. When a fibre population was absent in a subject, the value at the corresponding template fixel was assumed to be 0, thereby preserving one‐to‐one fixel correspondence across participants.

All fixel data *f* from each subject *j* were vectorised and concatenated into a matrix of size. For each FBA metric $Y_{\left(i,j,f\right)}$, based on the batch variable *i* (site, scanner model or protocol) and a design matrix of biological covariates *X*(*i,j*) (age, sex and ICV), the neuroCombat algorithm (Johnson et al. 2007) was applied to remove additive and multiplicative site (scanner model or protocol) effects while retaining biological variability. The ComBat harmonisation process involved modelling the diffusion metric for fixel as follows:

(1) $$Y_{\left(i,j,f\right)=\alpha_{\left(f\right)}+X_{\left(i,j\right)}\beta_{\left(f\right)}+\gamma_{\left(i,f\right)}+\delta_{\left(i,f\right)}\varepsilon_{\left(i,j,f\right)}}$$

where $\alpha_{\left(f\right)}$ is the overall FBA mean for fixel *f* and $\beta_{\left(f\right)}$ is the fixel‐specific vector of regression coefficients corresponding to $X_{\left(i,j\right)}$. The error terms $\varepsilon_{\left(i,j,f\right)}$ are assumed to follow a normal distribution with a mean of 0 and a variance of $\sigma_{f}^{2}$.

In the ComBat harmonisation framework, the parameters $\gamma_{\left(i,f\right)}^{*}$ and $\delta_{\left(i,f\right)}^{*}$ were estimated using an empirical Bayesian approach. The final harmonised FBA values are calculated as follows:

(2) $$Y_{\left(i,j,f\right)}^{\text{ComBat}}=\frac{Y_{\left(i,j,f\right)}-\left(\alpha_{\left(f\right)}^{*}+X_{\left(i,j\right)}\beta_{\left(f\right)}^{*}+\gamma_{\left(i,f\right)}^{*}\right)}{\delta_{\left(i,f\right)}^{*}}+\alpha_{\left(f\right)}^{*}+X_{\left(i,j\right)}\beta_{\left(f\right)}^{*}$$

The harmonised FBA values $Y_{\left(i,j,f\right)}^{\text{ComBat}}$ were reshaped back to fixel format and written to each subject's fixel image folder, enabling continuation of the FBA pipeline. For the ADNI dataset, harmonisation was performed separately for AD patients and CN controls.

### 2.6 Statistical Analysis

Whole‐brain FBA was performed using the MRtrix3 command *fixelcfestats* (Raffelt et al. 2015), which fits a GLM at each template‐defined fixel and performs connectivity‐based smoothing and statistical enhancement. For the TS dataset, a single‐group paired difference GLM was applied in each group, as the TS data were collected from the same individuals (Woolrich et al. 2009). For the ADNI dataset, a between‐group GLM was applied to compare AD patients with CN participants. Prior to harmonisation, the GLM included the scanner model, age, sex and ICV as nuisance covariates, and after harmonisation, age, sex and ICV were included as covariates. The output variable *EstimatedTotalCranialVol* obtained from T1‐weighted images in FreeSurfer version 6.0.0 (http://surfer.nmr.mgh.harvard.edu/fswiki) with the recon‐all pipeline was used as the ICV value (Dale et al. 1999).

For all whole‐brain FBA, statistical significance was assessed using non‐parametric permutation testing with 5000 permutations. Family‐wise error (FWE)‐corrected *p*‐values were calculated for each fixel, and significant fixels were identified at an FWE‐corrected threshold of *p* < 0.05 (Nichols and Holmes 2002). Regions containing significant fixels were visualised on the population tractogram using the *mrview* tool in MRtrix3.

For the ADNI dataset, tract‐level analyses were additionally performed on the fornix, as it exhibited significant alterations in FBA metrics following harmonisation. The left and right fornix bundles were automatically segmented using TractSeg (Wasserthal et al. 2018, 2019), and the mean FD, logFC and FDC values were extracted for each bundle. In addition, Cohen's *d* effect sizes (Cohen 1992) were calculated for the group differences (AD vs. CN) in the fornix region, both before and after ComBat harmonisation. Moreover, the relationships between these mean fornix FBA metrics and cognitive test scores, including the Mini‐Mental State Examination (MMSE) (Folstein et al. 1975) and the Montreal Cognitive Assessment (MoCA) (Nasreddine et al. 2005), were evaluated using ordinary least squares (OLS) regression models, adjusting for age, sex and ICV. In OLS regression, R (Raffelt et al. 2015) is a statistical measure that represents the proportion of variance in the cognitive score that can be predicted from the FBA metrics, and robustness is assessed by bootstrap resampling with 10,000 iterations (Efron 1979).

### 2.7 Data and Code Availability

Data used in the preparation of this article were obtained from the ADNI database (adni.loni.usc.edu). As such, the investigators within the ADNI contributed to the design and implementation of ADNI and/or provided data but did not participate in the analysis or writing of this report. A complete listing of ADNI investigators can be found at: http://adni.loni.usc.edu/wp‐content/uploads/how_to_apply/ADNI_Acknowledgement_List.pdf. TS data are available as a part of the Brain/MINDS Beyond human brain MRI project (https://hbm.brainminds‐beyond.jp). The ComBat harmonisation method used in this study was implemented using the *fixelcombat* command, which is available in the GitHub repository (https://github.com/remikamito/fixel_combat).

## 3 Results

### 3.1 Harmonisation in the TS Dataset

**FIGURE 1:**
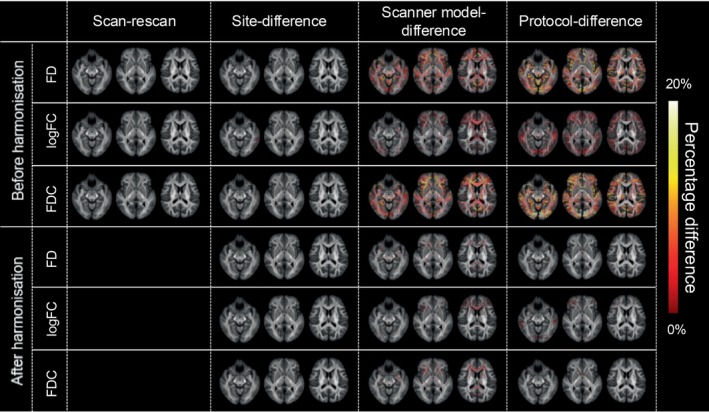
FBA of TS data before and after ComBat harmonisation. The percentage differences in FBA metrics (FD, logFC and FDC) with statistically significant differences (*p* < 0.05) in the whole brain. The percentage difference was calculated as follows: Percentage difference (%) = (Travelled FBA metric − Initial FBA metric) ÷ Initial FBA metric. The rows show these differences before and after ComBat harmonisation, while the columns represent the scan‐rescan condition, as well as comparisons across different sites, scanner models and protocols. The colour scale indicates the magnitude of the percentage difference (ranging from black to red‐yellow to white, indicating 0%–20%) in measurement bias within the white matter tracts. FD, fibre density; FDC, fibre density and cross‐section (equal to FD multiplied by FC); logFC, log‐transformed fibre bundle cross‐section.

To assess the measurement biases on FBA metrics (FD, logFC and FDC), we computed the magnitude of the percentage difference across different sites, scanner models and protocols using TS data before harmonisation, as shown in the upper part of Figure 1. No significant differences were observed in FD, logFC and FDC metrics for the scan‐rescan comparisons before harmonisation, suggesting that FBA metrics are stable under identical scanning conditions. However, substantial differences were observed when comparing data across different sites, scanner models and protocols before harmonisation. Specifically, slight differences were noted in partial areas between different sites, whereas more pronounced differences (8%–15%) were observed in FD, logFC and FDC metrics across different scanner models and protocols, highlighting considerable variability in FBA metrics when scanning conditions differ.

After applying ComBat harmonisation, all statistically significant differences in FD, logFC and FDC between different sites were eliminated, as illustrated in the lower part of Figure 1. Additionally, the differences across scanner models and protocols were substantially reduced post‐harmonisation. A small residual difference of 2%–3% persisted in the corpus callosum and frontal WM for scanner model‐related differences, whereas a similar difference of approximately 2%–3% remained in the cerebellum for protocol‐related differences. These results indicate that ComBat harmonisation effectively mitigates variability in FBA metrics introduced by differences in site, scanner model and protocol, thereby enhancing the comparability of multi‐centre dMRI studies.

### 3.2 Effectiveness of ComBat Harmonisation on FBA Metrics in Detecting AD‐Related Differences

**FIGURE 2:**
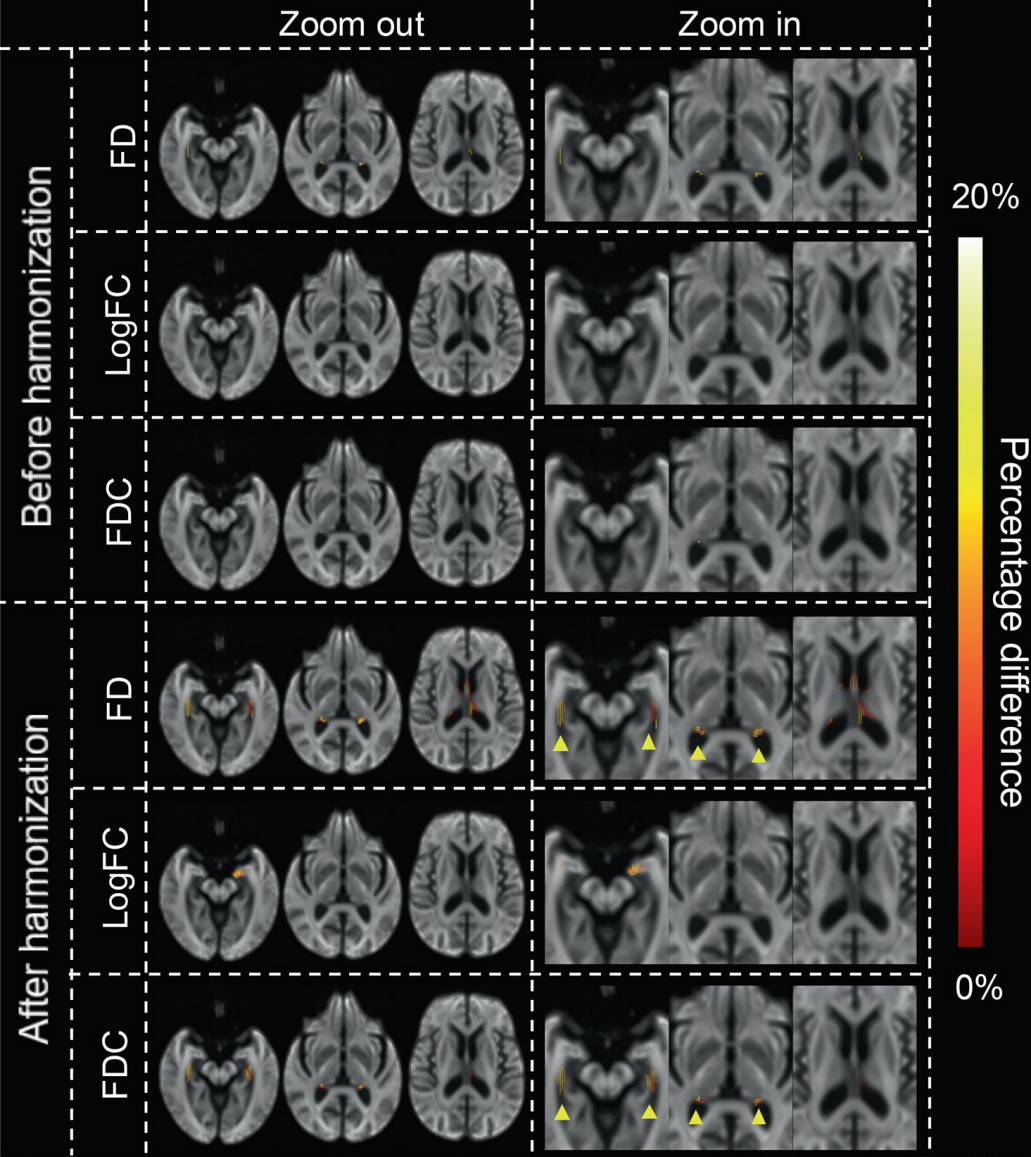
Differences in FBA metrics between AD patients and CN controls before and after ComBat harmonisation. The figure shows FD, LogFC and FDC metrics. The top three and bottom three rows represent pre‐harmonisation and post‐harmonisation results, respectively. The left side displays the ‘zoomed out’ view of the whole brain, whereas the right side shows the 1.5‐fold ‘zoomed in’ view of the fornix region. Before harmonisation, a limited significant reduction in FD in patients with AD compared to controls was detected (highlighted by yellow arrowheads). After harmonisation, the regions with significant reductions in FD in AD patients were more pronounced, particularly around the fornix region. Significant decreases in logFC and FDC also emerged after harmonisation. The colour bar represents the percentage difference, ranging from black to red‐yellow to white, indicating 0%–20%. The percentage difference marks the difference between AD patients and CN controls. FD, fibre density; FDC, fibre density and cross‐section (equal to FD multiplied by FC); logFC, log‐transformed fibre bundle cross‐section.

To determine whether ComBat harmonisation could enhance the detection of disease‐related changes by mitigating measurement bias in FBA metrics, we compared FBA metrics in the areas with significant reductions between data of AD patients and CN controls before and after harmonisation. As illustrated in Figure 2, prior to harmonisation, only a limited significant reduction in FD was observed in a small number of fixels in the fornix in data of AD patients compared with those of CN controls, and no significant differences were observed for logFC or FDC metrics.

After applying ComBat harmonisation to adjust for MRI scanner effects, the region with significant FD reduction in the fornix in patients with AD became more extensive and clearly defined. Additionally, significant reductions in logFC and FDC were observed in the same region, demonstrating the improved detection of disease‐specific alterations after harmonisation.

### 3.3 Tract‐Level Analysis and Correlations With Cognitive Scores in Fornix

**FIGURE 3:**
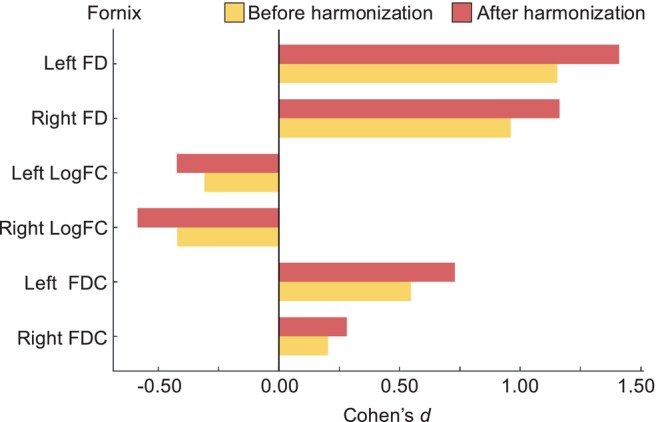
Cohen's *d* effect sizes in the fornix before and after ComBat harmonisation. Cohen's *d* values for the FBA metrics, including FD, logFC and FDC, in the left and right fornix are displayed. Yellow bars represent effect sizes before harmonisation, and red bars represent effect sizes after harmonisation. The vertical line at 0.00 indicates no effect. FD, fibre density; FDC, fibre density and cross‐section (equal to FD multiplied by FC); logFC, log‐transformed fibre bundle cross‐section.

To further examine the relationship between FBA metrics and cognitive function, we calculated the average FD, logFC and FDC values for the whole brain, as well as the left and right fornix. Cohen's *d* effect sizes between AD and CN groups increased after ComBat harmonisation (Figure 3), indicating that harmonisation enhanced sensitivity to disease‐related alterations in fixel‐based metrics.

We then assessed correlations between these metrics and the cognitive scores from MMSE (Table 5) and MoCA (Table 6) before and after harmonisation. No significant correlations were found between whole‐brain average FD, FDC or LogFC and MMSE scores before or after harmonisation. However, the average FD of the fornix showed a significant positive correlation with MMSE scores before harmonisation (left fornix: coefficient = 24.695, *p* < 0.001, *R* ^2^ = 0.334, 95% confidence intervals [CIs] = [0.216–0.540]; right fornix: coefficient = 22.132, *p* < 0.005, *R* ^2^ = 0.292, 95% CI = [0.162–0.513]). Interestingly, these correlations were further strengthened after harmonisation (left fornix: coefficient = 33.669, *p* < 10^−5^, *R* ^2^ = 0.420, 95% CI = [0.293–0.610]; right fornix: coefficient = 30.769, *p* < 10^−4^, *R* ^2^ = 0.370, 95% CI = [0.228–0.583]). In addition, after harmonisation, the average FDC of the left fornix showed a statistically significant positive correlation with MMSE scores (coefficient = 21.003, *p* < 0.05, *R* ^2^ = 0.209, 95% CI = [0.093–0.414]), although no significant correlation was found between the average FDC of the right fornix and MMSE scores before harmonisation (coefficient = 12.365, *p* = 0.141, *R* ^2^ = 0.160, 95% CI = [0.070–0.373]). Similar patterns were observed with MoCA scores, where correlations with fornix metrics consistently increased after harmonisation. Bootstrap resampling confirmed that *R* ^2^ confidence intervals shifted upward following harmonisation, supporting the robustness and enhancement of the associations between FBA metrics in the fornix and cognitive scores (Tables 5 and 6). These findings demonstrate that ComBat harmonisation facilitates the detection of AD‐related differences in FBA metrics, particularly in the fornix region, and strengthens the association of these metrics with cognitive function.

**TABLE 5:** The correlation of MMSE and FBA metrics.

Region	FBA	Before harmonisation				After harmonisation			
		Coefficient	*p*	*R* ^2^	95% CI	Coefficient	*p*	*R* ^2^	95% CI
Whole brain	FD	−35.290	0.175	0.155	0.069–0.383	−22.066	0.585	0.129	0.059–0.345
	logFC	33.535	0.081	0.175	0.050–0.346	23.878	0.096	0.170	0.057–0.367
	FDC	−2.006	0.929	0.124	0.079–0.387	21.439	0.313	0.141	0.073–0.383
Left fornix	FD	24.695	**0.000**	0.334	0.216–0.540	33.669	**0.000**	0.420	0.293–0.610
	logFC	−8.191	**0.023**	0.209	0.113–0.450	−10.887	**0.012**	0.226	0.121–0.459
	FDC	12.365	0.141	0.160	0.070–0.373	21.003	**0.023**	0.209	0.093–0.414
Right fornix	FD	22.132	**0.001**	0.292	0.162–0.513	30.769	**0.000**	0.370	0.228–0.583
	logFC	−8.443	**0.010**	0.231	0.127–0.481	−11.620	**0.003**	0.263	0.148–0.503
	FDC	0.220	0.972	0.124	0.052–0.327	4.894	0.491	0.132	0.058–0.347

*Note:* Bold font indicates statistical significance.

Abbreviations: CI, confidence interval; FBA, fixel‐based analysis; FD, fibre density; FDC, fibre density and cross‐section; logFC, log‐transformed fibre bundle cross section; MMSE, Mini‐Mental State Examination.

**TABLE 6:** The correlation of MoCA and FBA metrics.

Region	FBA	Before harmonisation				After harmonisation			
		Coefficient	*p*	*R* ^2^	95% CI	Coefficient	*p*	*R* ^2^	95% CI
Whole brain	FD	−58.927	0.136	0.140	0.053–0.371	−32.868	0.593	0.107	0.038–0.348
	logFC	37.605	0.203	0.130	0.038–0.335	38.235	0.079	0.155	0.039–0.392
	FDC	−16.465	0.631	0.106	0.043–0.345	34.093	0.292	0.121	0.056–0.381
Left fornix	FD	29.677	**0.004**	0.236	0.121–0.465	42.011	**0.000**	0.306	0.179–0.521
	logFC	−9.663	0.082	0.154	0.064–0.389	−11.834	0.079	0.155	0.065–0.384
	FDC	12.502	0.332	0.118	0.041–0.354	24.727	0.081	0.154	0.059–0.398
Right fornix	FD	27.361	**0.009**	0.215	0.092–0.473	39.345	**0.001**	0.280	0.142–0.520
	logFC	−10.783	**0.033**	0.179	0.086–0.422	−14.080	**0.021**	0.192	0.089–0.429
	FDC	−1.683	0.860	0.102	0.035–0.318	4.761	0.661	0.105	0.034–0.341

*Note:* Bold font indicates statistical significance.

Abbreviations: CI, confidence interval; FBA, fixel‐based analysis; FD, fibre density; FDC, fibre density and cross‐section; logFC, log‐transformed fibre bundle cross section; MoCA, Montreal Cognitive Assessment.

## 4 Discussion

In this study, we performed ComBat harmonisation within the whole‐brain FBA framework and demonstrated its effectiveness in reducing measurement bias by minimising site, scanner and protocol‐related differences in derived measures using a substantial TS dataset. We also examined the effectiveness of this harmonisation approach to identify AD‐relevant changes in the ADNI dataset. Here, we demonstrated pronounced AD‐related differences in the fornix post‐harmonisation, which were minimally observed before harmonisation. These findings underscore the effectiveness of ComBat harmonisation in enhancing the reliability of FBA metrics.

### 4.1 ComBat Harmonisation in TS Data

The variability in FBA metrics due to differences in scanner models and protocols was approximately 8%–15%, which is comparable to the 5%–10% variability reported for DTI and NODDI metrics in previous studies (Andica et al. 2020; Chung et al. 2016). This highlights an important feature of FBA metrics: while they offer superior accuracy in evaluating crossing fibres compared to voxel‐based metrics, they are more sensitive to differences in acquisition. As FBA relies on the CSD model, which is an advanced dMRI analysis approach that requires high angular resolution diffusion imaging data (Tournier et al. 2007; Behrens et al. 2007), which is difficult to reproduce, emphasising the importance of harmonisation techniques for ensuring reliability in multi‐centre studies.

Our findings confirm that FBA metrics are stable under identical scanning conditions, while they are significantly affected by site, scanner model and protocol differences. ComBat harmonisation provides a robust and effective solution to these effects. Specifically, ComBat effectively reduced inter‐site variability from 8%–15% to 2%–3%, markedly improving the comparability of FBA metrics across multi‐centre datasets.

Importantly, when comparing these harmonisation‐induced reductions in variability with disease‐related effect sizes, we found that the pre‐harmonisation measurement bias (8%–15%) was comparable to or even larger than the disease‐specific differences reported in previous FBA studies. For example, differences in FD, FC and FDC values were approximately 10%–20% for ad (Mito et al. 2018), 5%–10% for Parkinson's disease (Andica et al. 2023, 2021) and 3%–10% for major depressive disorder (Lyon et al. 2019) and chronic schizophrenia (Grazioplene et al. 2018). This underscores the necessity for harmonisation in multi‐centre FBA studies, as the inherent variability introduced by differences in acquisition conditions could obscure meaningful disease‐specific alterations. By applying ComBat harmonisation, we effectively reduced this variability, thereby enhancing the ability to detect biologically relevant changes in FBA metrics.

### 4.2 ComBat Harmonisation in ADNI Data

To further validate whether the detection of disease‐specific changes is enhanced after harmonisation, we applied harmonisation to the ADNI dataset, which included data from patients with AD and CN controls. In the present study, dMRI data were acquired from six different MRI scanners. Prior to harmonisation, only limited reductions in FD were detected in the exceedingly small fornix area in patients with AD compared to CN controls, with no significant differences observed for logFC or FDC metrics. This suggests that the inherent variability introduced by different scanners can mask subtle, yet clinically relevant, structural changes in the brain. After applying ComBat harmonisation to adjust for MRI scanner effects, the region showing significant FD reduction in the fornix of patients with AD became more extensive and clearly defined, which is consistent with prior studies utilising a single MRI scanner (Mito et al. 2018; Pievani et al. 2010; Oishi and Lyketsos 2014; Nowrangi and Rosenberg 2015). Therefore, the finding that ComBat harmonisation properly defined FD reduction in the fornix highlights the utility of this harmonisation technique in reducing inter‐scanner variability and uncovering disease‐specific alterations that may otherwise go unnoticed.

As we know, the fornix originates from the alveus of the hippocampus, which continues caudally into the fimbria. The fimbriae become the crura of the fornix, which converge posteriorly to form the body of the fornix. Previous studies have shown that the integrity of the fornix is crucial for cognition, and reduced fornix integrity has been shown to predict memory decline and progression to AD (Mielke et al. 2012; Wang et al. 2020). Therefore, our study also assessed the correlation between FBA metrics in the fornix and cognitive performance. Before harmonisation, significant correlations were observed between FD and cognitive scores (MMSE and MoCA), and these correlations remained statistically significant post‐harmonisation. Notably, significant correlations were not observed between FDC and MMSE before harmonisation; however, they were observed after harmonisation. The increased post‐harmonisation *R* ^2^ values indicate that scanner‐related variance, which had previously masked true biological differences, was effectively mitigated. Moreover, the 95% CI obtained by bootstrap resampling (10,000 iterations) of *R* ^2^ values consistently excluded 0, and both the lower and upper bounds shifted upward after harmonisation, indicating not only robustness but also an enhanced strength of the reported correlations. This enhancement in correlation strength further supports the argument that harmonisation is a critical step in ensuring the reliability of multi‐centre neuroimaging studies, particularly in the context of subtle changes associated with neurodegenerative diseases.

### 4.3 Limitations and Future Directions

As ComBat employs a Bayesian framework to estimate measure acquisition‐specific effects, it requires an adequate number of samples per group for a stable estimation. Given that some scanners have < 10 samples, the harmonisation process may yield numerically valid but potentially unstable results. The group with site‐difference data (*n* = 10) was considerably smaller compared to the groups with the MRI scanner model (*n* = 23) and protocol differences (*n* = 31). This imbalance may have reduced the statistical power to detect site‐related differences, warranting cautious interpretation of these results. Notably, in the comparison between AD and CN controls, data from 56 subjects were used to balance participants across MRI scanners. The small sample size limits the ability to detect subtle differences and generalise findings in ADNI data. Future studies with larger and more evenly distributed sample sizes across comparison groups are crucial to further examine the usefulness of harmonisation. Moreover, some previous studies have reported that ComBat harmonisation did not markedly alter group differences or associations with cognition when applied to structural MRI or conventional diffusion metrics (Zavaliangos‐Petropulu et al. 2019; Tassi et al. 2024). In contrast, our results demonstrated that ComBat substantially enhanced the detection of disease‐related alterations in fixel‐based metrics, particularly within the fornix, and strengthened their correlations with cognitive measures. This discrepancy may reflect differences in the imaging features examined (fixel‐wise vs. voxel‐ or ROI‐based measures) and highlights the unique sensitivity of FBA to subtle WM alterations.

Furthermore, the study utilised single‐shell CSD in ADNI data, which can overestimate WM volume in voxels containing GM or CSF, contributing to variability. To improve accuracy, future research should adopt multi‐shell acquisitions, as these provide more comprehensive diffusion information and enable more precise modelling of complex tissue structures. Ensuring consistency in data acquisition and employing multi‐shell approaches would significantly enhance the reliability and reproducibility of findings.

Another limitation is that we focused only on AD and CN groups in the present study. Given that FBA metrics are sensitive to subtle microstructural changes, future work should extend harmonisation‐based analysis to individuals with mild cognitive impairment, which would provide further insight into the progressive trajectory of WM alterations across the AD spectrum.

Harmonisation is essential for multi‐centre neuroimaging studies, especially for complex disorders like AD. By minimising measurement biases, techniques like ComBat help in detecting subtle disease‐related differences, increasing statistical power and generalisability. This also facilitates the identification of consistent biomarkers across different imaging platforms, aiding clinical translation. ComBat is a statistical batch harmonisation technique that has become popular in multi‐centre neuroimaging studies, potentially because it can be implemented retrospectively on post‐processed data, making it easy to adopt. However, numerous other harmonisation techniques are used, particularly for dMRI data, such as rotational invariant spherical harmonics (Mirzaalian et al. 2015, 2018) and deep learning‐based approaches (Hu et al. 2023). While this study focused on the ComBat method, future research should systematically compare its effectiveness with alternative techniques to identify the most suitable approach for specific datasets and study designs.

## 5 Conclusion

ComBat harmonisation effectively mitigates the measurement bias in FBA metrics introduced by site, scanner model and protocol variations, indicating its potential to detect subtle changes in neuropsychiatric disorders. These results underscore the potential of harmonisation to enhance the reliability and reproducibility of multi‐centre dMRI studies, supporting a transition from single‐centre studies with limited sample sizes to large‐scale, multi‐centre dMRI investigations with greater statistical power.

## Ethics Statement

The local ethics committee approved this study.

## Consent

All participants provided written informed consent.

## Conflicts of Interest

The authors declare no conflicts of interest.

## Data Availability

The data that support the findings of this study are available in the GitHub repository at https://github.com/remikamito/fixel_combat. These data were derived from the following resources available in the public domain: Alzheimer's Disease Neuroimaging Initiative, https://adni.loni.usc.edu/. Brain/MINDS Beyond human brain MRI project, https://hbm.brainminds‐beyond.jp.
